# Interior renovation of a general practitioner office leads to a perceptual bias on patient experience for over one year

**DOI:** 10.1371/journal.pone.0193221

**Published:** 2018-02-20

**Authors:** Jérôme Gauthey, Raphaël Tièche, Sven Streit

**Affiliations:** 1 Spitalzentrum Biel, Biel, Switzerland; 2 General Practice Office, Grenchen, Switzerland; 3 Institute of Primary Health Care (BIHAM), University of Bern, Bern, Switzerland; Dartmouth-Hitchcock Medical Center, UNITED STATES

## Abstract

**Introduction:**

Measuring patient experience is key when assessing quality of care but can be biased: A perceptual bias occurs when renovations of the interior design of a general practitioner (GP) office improves how patients assessed quality of care. The aim was to assess the length of perceptual bias and if it could be reproduced after a second renovation.

**Methods:**

A GP office with 2 GPs in Switzerland was renovated twice within 3 years. We assessed patient experience at baseline, 2 months and 14 months after the first and 3 months after the second renovation. Each time, we invited a sample of 180 consecutive patients that anonymously graded patient experience in 4 domains: appearance of the office; qualities of medical assistants and GPs; and general satisfaction. We compared crude mean scores per domain from baseline until follow-up. In a multivariate model, we adjusted for patient’s age, gender and for how long patients had been their GP.

**Results:**

At baseline, patients aged 60.9 (17.7) years, 52% females. After the first renovation, we found a regression to the baseline level of patient experience after 14 months except for appearance of the office (p<0.001). After the second renovation, patient experience improved again in appearance of the office (p = 0.008), qualities of the GP (p = 0.008), and general satisfaction (p = 0.014). Qualities of the medical assistant showed a slight improvement (p = 0.068). Results were unchanged in the multivariate model.

**Conclusions:**

Interior renovation of a GP office probably causes a perceptual bias for >1 year that improves how patients rate quality of care. This bias could be reproduced after a second renovation strengthening a possible causal relationship. These findings imply to appropriately time measurement of patient experience to at least one year after interior renovation of GP practices to avoid environmental changes influences the estimates when measuring patient experience.

## Introduction

Measuring patient experience in primary care is one of the main health care objectives in Europe in the last decade with the purpose to globally improve quality of care [1–3]. Recent reviews had shown that the measurement of patient experience is as important as clinical effectiveness and patient safety [4–5]. Good patient experience is associated with an improvement in patient’s adherence [4], increase in patient’s use of screening service [4] and reduction in patient’s use of emergency service [4].

Patient experience is known as the “sum of all interactions, shaped by an organization’s culture that influence patient perceptions across the continuum of care” [6]. It depends mainly on patient expectations before a consultation and if these expectations were perceived to be met successfully during the consultation [7]. Thus, the most important factor for patient experience is the doctor-patient communication [8–9]. These performance appraisals, like any other perceptions, are known to be subject to perceptual bias [10].

The most common perceptual bias in performance appraisal is the halo effect [10], where a single characteristic influences the entire evaluation of other characteristics. For example: a change of a purely visual architecture appearance is leading to a temporary better perception of all other domains of the patient experience [11]. In hospital care, the influence of the environment is known as an important factor, as reflected by a study which found a clinical impact on recovery from surgery depending on the window view of patients [12]. A recent review summarized evidence of the impact of physical environment on staff, patients and their families in hospital care [13], whereas the influence of the physical environment in primary care is not well studied so far. Healthcare design influence patient experience using reducing errors by standardizing the examination room and using increasing communication and well-being by adjusting the lighting or by increasing comfort (visual, acoustic, furniture) [13].

Only two previous studies explored the importance of the visual environment on the patient experience in primary care. In a previous study, we could identify patient experience to be significantly improved due to a recent renovation of a single General Practitioner (GP) [14] (including patient overall experience and patients’ perception of GP skills), but we did not assess the duration of this halo effect. In another study, the halo effect was found to last more than 11 months for staff satisfaction when a GP surgery moved from old premises to new ones [15]. There is a need to understand the duration of this halo effect in order to correctly interpret findings from currently performed patient experience assessments [1–3]. The new premises decreased patient’s anxiety and improved patient’s satisfaction as well as the perceived communication skills of the GP [15].

## Material and methods

### Study design

This is a prospective observational study that assessed patient experience using a paper questionnaire at baseline and three times during a 2-year follow-up. For all time points, a sample of consecutive patients was invited to reply anonymously to the questionnaire. During the observation period, the GP office was renovated twice.

### Context

Our study was conducted in a single GP office funded 1981 in Grenchen, a town with 17,000 inhabitants in Switzerland [16]. The population of the GP office was semi-urban, with 53% female patients registered, aged 49 years in mean and mostly German speaking (>80%). During the observation period from April 2014 to June 2016, 2,083 patients had 14,065 consultations at the GP office.

The office was run by GP1 until 2013, when another GP joined the office (GP2). In addition, three GP trainees worked under supervision at the GP office for some months.

The team of five female medical assistants changed only once during the observation period: an employee working three days a week was replaced by a medical assistant working 2 days/week. No intervention was done on the physical appearance of the staff and no special training took place to improve the skills of the medical assistants.

### Renovation and modernization of the GP office

The transition of the GP office into a group GP office led to the decision to plan two renovations in order to modernize the office which was renovated last in 1981, and to provide more space for the larger team to work.

A first renovation was carried out from June to August 2014 as described previously [14]. In summary, entry hall, reception area, blood sampling room, waiting room, staff room, and pharmacy were painted in white/blue and refurnished (Fig 1 in [14]). The refurbishment included a new lightning system, new artwork, a new air-conditioning system in the waiting room and entry hall and some new furniture. No changes were made in other known aspect to influence patient experience like seating comfort, seating patterns or equipment standard in the consultation rooms, the air smell/quality, and neither were measurements taken to improve the view out of the windows or to decrease overhearing conversations from the reception at the waiting room.

**Fig 1:**
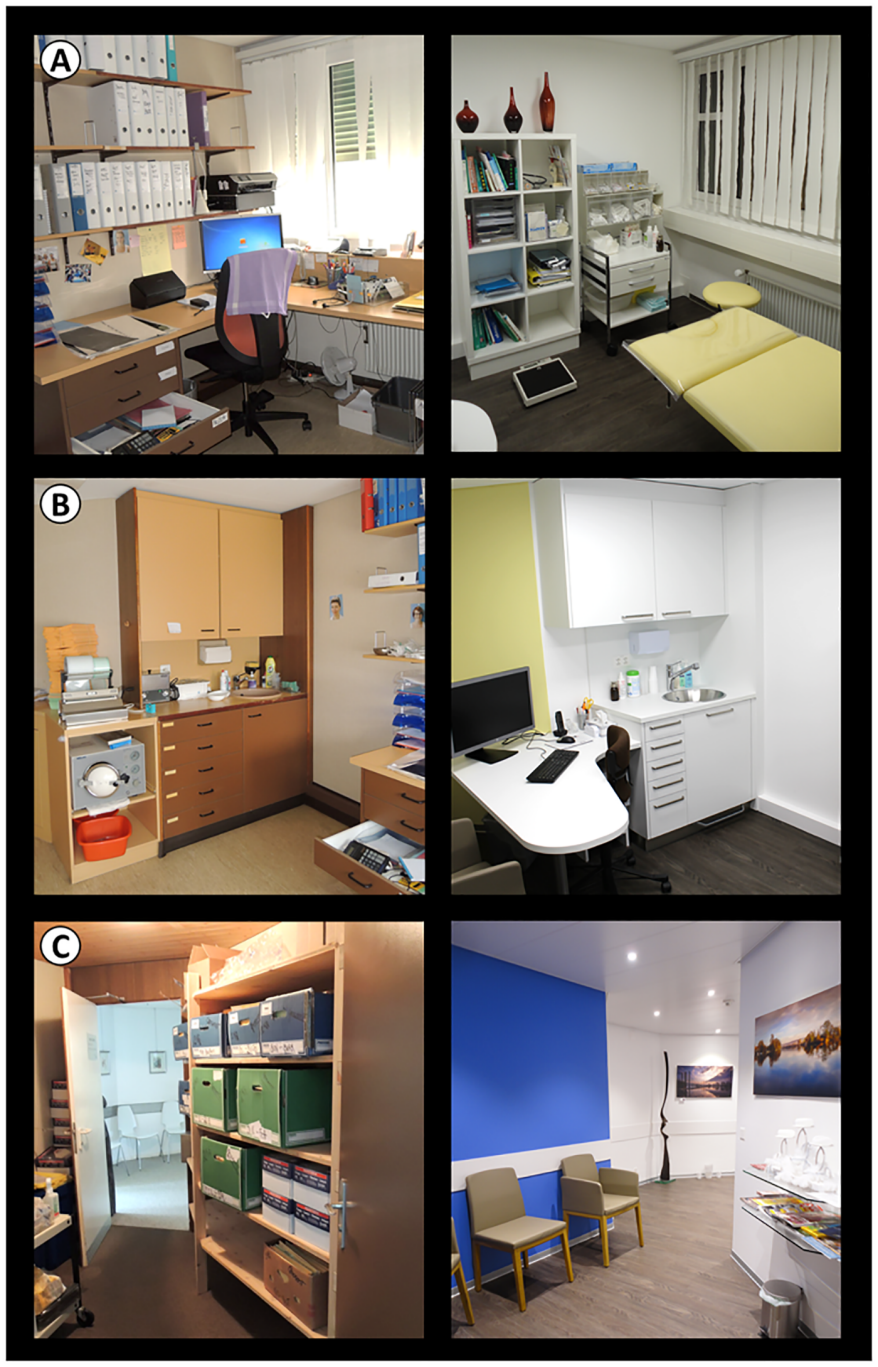
Pictures before and after the second renovation in a GP office. (A+B) Former secretary working space was replaced by a fifth treatment room. (C) A second waiting area for the patients was constructed where a written medical history storage room was used previously.

The second renovation took place from February to March 2016. The floors were newly adapted to make the rooms wheelchair accessible, the corridors and the four treatment rooms were painted, one was refurnished and the lightning concept was adapted. A former secretary working space and storage room was replaced by a fifth treatment room (Panel A+B, Fig 1). A second waiting area for the patients was constructed where a written medical history storage room used to be (Panel C, Fig 1).

### Patients and intervention

**Fig 2:**
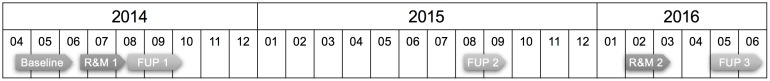
Time line of the study. R&M = Renovation and modernization; FUP = follow-up.

One year after GP2 joined the GP office, a paper questionnaire was distributed to a consecutive sample of patients two months before the first renovation (baseline) as well as two months (FUP1) and 14 months after renovation (FUP2). The time point of FUP2 was chosen to be six months before the second renovation took place. A final follow-up was done three months after the second renovation (FUP3). Fig 2 provides an overview timeline.

At all four time points, a sample of 180 consecutive patients was invited to participate. The questionnaire was distributed by the medical assistants to all consecutive patients when they arrived for an appointment to the GP office on the day of the follow-up. Oral informed consent was obtained and the patients replied anonymously. The filled-out questionnaire was collected before the patient left the office. GPs did not interfere with the distribution of the questionnaire. Patients >18 years of age who were able to communicate in German were included. Patients consulting for an emergency or consulting for the first time at the GP office were excluded. The same questionnaire was distributed at FUP1, FUP2 and FUP3.

We aimed for a sample size of 150 replies per questionnaire based on the fact that a national questionnaire to study patient experience in Switzerland is usually handed out to 150 patients [1–3, 14] and that in our previous study the equal sample size resulted in sufficient data to detect statistically significant differences in patient experience before and after renovation [14]. Expecting a response rate of 80%, we aimed to distribute 180 questionnaires. However, we handed out too many copies of the questionnaire at FUP2, and received 210 responses. To avoid selection bias, we kept all 210 responses for further analysis.

### Measurements

As described previously [14], we used a questionnaire in line with commonly used to assess patient satisfaction in Switzerland [1–3, 17]. We measured patient experience using a questionnaire with 12 specific items and grouped the items in four domains using Cronbach’s alpha to assess the correlation of the items within the same domain resulting in a good Cronbach’s alpha ranging from 0.75 to 0.88 per domain (S1 Appendix). The following items (domain) were used for the analysis: 1) Appearance of the facility; 2) diagnostic equipment; 3) level of hygiene; 4) prompt response to patient needs (i.e. timely manner to get an appointment with the GP); and 5) punctuality and dependability of the staff (domain: appearance of the office). 6) Dress and grooming of the medical assistants; and 7) friendliness and courtesy of the medical assistant (domain: qualities of the medical assistant). 8) Attentiveness and responsiveness of the GP to patient needs; 9) GP’s perceived level of expertise; 10) GP’s level of empathy (domain: qualities of the GP). 11) Medical performance of the GP office in general; and 12) overall satisfaction with the office (domain: general satisfaction). We asked patients to grade each item on a Likert scale from 1 (very poor) to 6 (very good). In addition, we asked participants for their age, gender, assigned GP and duration of that assignment (S1 Dataset).

### Ethical considerations

The study was conducted in compliance with Swiss data protection and privacy laws. Since no health-related data were collected and patients replied anonymously, additional approval by an ethics committee was not required [18]. Each Patient was made aware that their replies will be used for a study and informed that non-participation would not result in different treatment. Patients gave oral consent.

### Statistical analysis

For descriptive analysis, we calculated means and standard deviations (SD), or used numbers and proportions. We compared continuous data using p-for trend and categorical data using Chi2-test.

We calculated means and 95% confidence intervals (CI) of all four time points for each of the four domains using a univariate regression model with robust standard errors to account for the possibility of repeated measurements within the same individuals. We further adjusted for patient age and gender, GP assignment and duration of GP assignment using a multivariate linear regression model using robust standard errors. We restricted the analysis on baseline, FUP1 and FUP2 to assess the duration of the cognitive bias and in a second model to FUP2 and FUP3 to assess reproducibility of the cognitive bias after a second renovation. For all analyses, we used STATA release 14.2 (Stata Corp, College Station, TX, USA). We set the level of significance at a two-sided p<0.05.

## Results

A total of 152 patients (response rate 84%) completed the questionnaire at baseline, 169 (94%) at FUP1, 210 (91%) at FUP2, and 161 (89%) at FUP3.

Table 1 depicts patient characteristics: At baseline, patients’ mean age was 60.9 (SD 17.7) years, 52% were female. During follow-up, patients’ age and gender did not change significantly. However, the GP assignment did change most probably as a consequence of GP1 working less, and GP2 working more working days per week (p<0.001).

**Table 1:** Patient characteristics at baseline and at each follow-up (FUP).

Patient characteristics	Baseline (n = 152)	FUP 1 (n = 169)	FUP 2 (n = 210)	FUP 3 (n = 161)	P-value^a^
**Age**, years (SD)	60.9 (17.7)	57.3 (17.9)	56.7 (19.0)	59.1 (18.1)	0.36
**Women**, n (%)	77 (52.0)	93 (55.7)	105 (51.2)	85 (54.5)	0.82
**GP assignment**, n (%)					<0.001
GP1	81 (56.3)	83 (50.6)	81 (39.9)	44 (27.9)	
GP2	45 (31.3)	59 (36.0)	80 (39.4)	87 (55.1)	
Other assignment^b^	18 (12.5)	22 (13.4)	42 (20.7)	27 (17.1)	

^a^ P for trend for continuous data, chi2-test for categorical data

^b^ Assigned to GP3, a combination of GPs, or a combination of GP and GP trainee

### Crude patient experience at baseline and during follow-up

**Fig 3:**
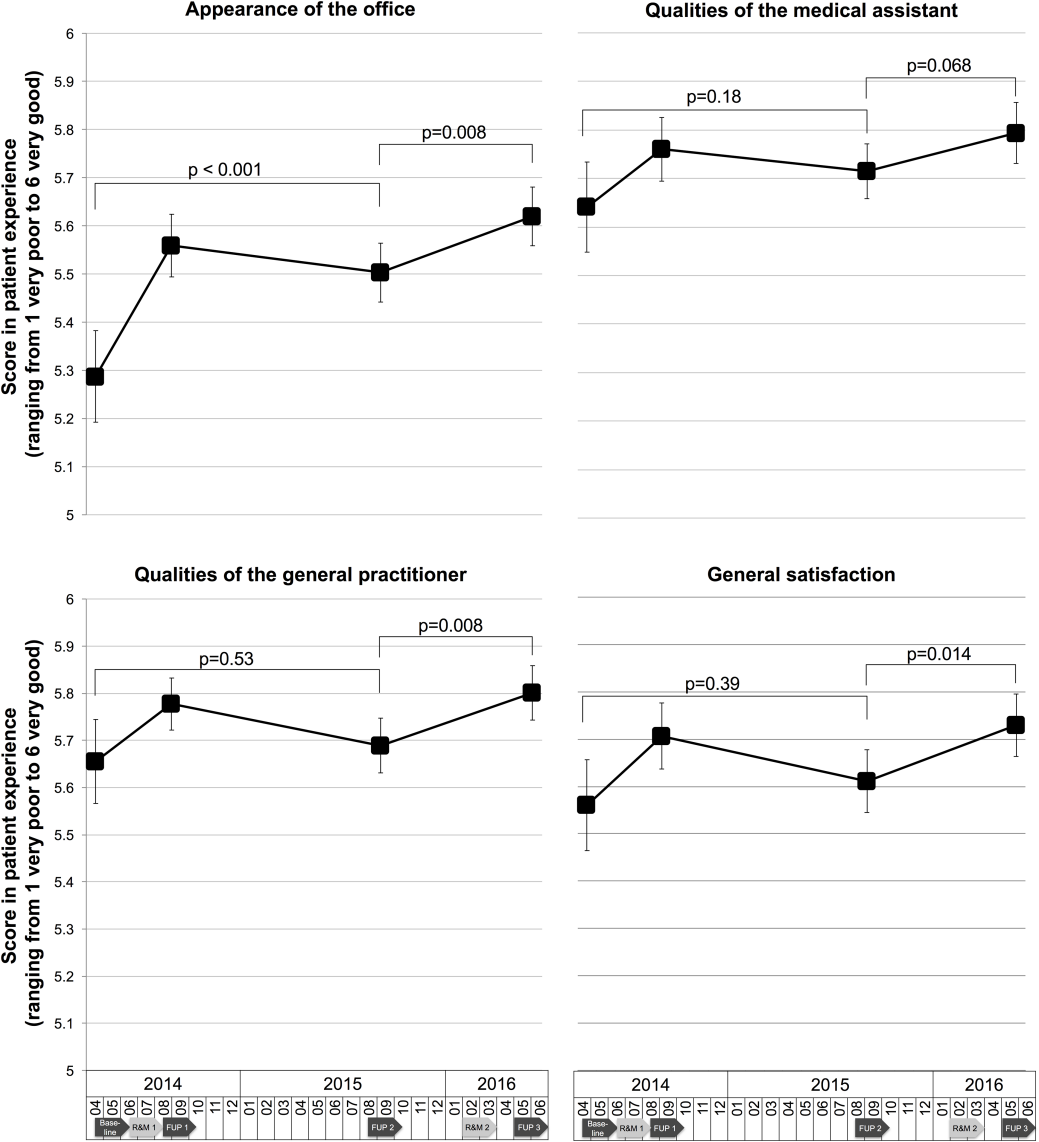
Patient experience in four domains at baseline and during follow-up (unadjusted). Y axis restricted to a score from 5 to 6. X axis represents time in months. P-values from univariate regression models. R&M = renovation and modernization, FUP = follow-up.

Fig 3 summarizes the crude patient experience (measured on a scale from 1 (very poor) to 6 (very good) per domain at baseline and during a follow-up of 3 years. As described previously, all domains improved after the first renovation [14]. At FUP 2, 14 months after the first renovation, patient experience was still improved regarding the appearance of the GP office (p<0.001). However, the other three domains showed no significant difference from baseline (p between 0.18 to 0.53), thus the perceptual bias had disappeared.

FUP 2 was also the new baseline for the second renovation and FUP 3 the assessment 3 months after the second renovation. Again, an improvement in appearance of the office (p = 0.008), qualities of the general practitioner (p = 0.008), and general satisfaction (p = 0.014) was found. The perceptual bias was reproduced. Qualities of medical assistants, only showed a slight improvement (p = 0.068).

### Duration of perceptual bias on patient experience after first renovation

Table 2 shows the adjusted patient experience before and after the first renovation per domain. We found an already very high overall satisfaction (score ranging from 5.07 to 5.50) before the first renovation. Fourteen months after the first renovations (FUP2), the adjusted patient experience was still significantly higher than at baseline for the appearance of the office (change from baseline score 0.21, p for trend <0.001). The other three domains showed a regression to the baseline level (change from baseline, p for trend): Qualities of medical assistant (0.09, p = 0.17), qualities of general practitioner (0.05, p = 0.46), and general satisfaction (0.06, p = 0.38).

**Table 2:** Duration of perceptual bias on patient experience after first renovation (adjusted^a^).

Domains	Baseline 2 months before 1^st^ renovation Mean^b^ (95%CI)	FUP 1 Month 2 after renovation Increase^b^ (95%CI)	FUP 2 Month 14 after renovation Increase^b^ (95%CI)	P for trend
Appearance of the office	5.07 (4.81–5.33)	+0.23 (0.12–0.34)	+0.21 (0.10–0.32)	<0.001
Qualities medical assistant	5.46 (5.22–5.69)	+0.12 (0.004–0.24)	+0.09^c^ (-0.03–0.20)	0.17
Qualities general practitioner	5.50 (5.29–5.70)	+0.11 (0.01–0.21)	+0.05^c^ (-0.06–0.15)	0.46
General satisfaction	5.26 (5.00–5.51)	+0.12 (0.004–0.23)	+0.06^c^ (-0.06–0.17)	0.38

^a^ Adjusted for patient age, gender, GP assignment and duration of GP assignment

^b^ On a scale from 1 (very poor) to 6 (very good), thus e.g. 0.23 means an increase by 0.23 on that scale after renovation

^c^ No statistically significant increase from baseline (p>0.05)

### Reproducible perceptual bias on patient experience after second renovation

The effect of the second renovation on the adjusted patient experience is shown in Table 3. Three months after the second renovation (FUP 3), all domains improved again with one exception: the qualities of medical assistants showed only a slight increase in patient experience. Most improvement was found in general satisfaction (change 0.11, p = 0.021) and appearance of the office (change 0.10, p = 0.031). Also, the qualities of the general practitioner showed significant improvement (change 0.09, p = 0.034). The quality of the medical assistants was already the highest of domains before the second renovation (scoring 5.47 on scale of maximal 6) and showed only a tentative improvement three months after the second renovation (change 0.08, p = 0.092).

**Table 3:** Reproducible perceptual bias on patient experience after second renovation (adjusted^a^).

Domains	FUP2 6 months before 2^nd^ renovation Mean^b^ (95%CI)	FUP3 Month 3 after 2^nd^ renovation Increase^b^ (95%CI)	P-value
Appearance of the office	5.14 (4.89–5.38)	+0.10 (0.09–0.19)	0.031
Qualities medical assistant	5.47 (5.23–5.71)	+0.08 (-0.01–0.17)	0.092
Qualities general practitioner	5.39 (5.15–5.62)	+0.09 (0.01–0.17)	0.034
General satisfaction	5.18 (4.91–5.46)	+0.11 (0.02–0.21)	0.021

^a^ Adjusted for patient age, gender, GP assignment and duration of GP assignment

^b^ On a scale from 1 (very poor) to 6 (very good), thus e.g. 0.10 means an increase by 0.10 on that scale after renovation

## Discussion

### Summary

In a GP office that was renovated, we observed a perceptual bias of patient experience that improved how patients assessed quality of care in our study. After 14 months, patient experience was still improved regarding the appearance of the office while qualities of GPs, medical assistants and overall satisfaction showed a regression to the mean. A second renovation reproduced the same perceptual bias in all four domains again. The findings strengthen the possible causal relation of renovation on patient experience and the confounding role of perceptual bias on other domains such as quality of care by GPs and assistants and furthermore indicate that this halo effect is wearing off after approximately one year.

### Limitations and strengths

Our study has several possible limitations to mention. First, we did not use a unique identifier at baseline that would have made it possible to follow up the same patients over time and use the design of a cohort study. However, we found it more straightforward to invite consecutive patients and measure patient experience of those attending the GP office at the time of the follow-up. Second, we developed quality measures that were not validated yet but are in daily use in Switzerland when measuring patient experience. Due to the high correlation coefficients (Cronbach’s alpha ranging from 0.75 to 0.88) we considered our 12 quality indicators are grouped well according to their content validity. Third, we don’t know if and how many patients participated in more than one questionnaire. Thus, we had to decide on the choice of paired or non-paired analysis. We chose the latter design and used a robust standard error in the regression models to account for this fact. Fourth, we observed an already high satisfaction in patient experience before the first renovation (scoring above 5 on a scale to maximum 6). However, we were still able to detect a significant improvement in patient experience after the second renovation except for qualities of medical assistants, but this domain was rated highest before the second renovation. Thus, a ceiling effect could limit the size of improvement in this quality domain [19]. The impossibility to improve the environment of the office during 3 years without affecting other aspect of the patient experience is the main difficulty for such a study. However, that is the strength of our study to limit the analysis to one GP office. Further, the high response rate (about 90% or more) in all 4 questionnaires strengthen our generalizability to the target population of all patients of this GP office. As we chose a typical GP office in Switzerland in respect to gender and age distribution of the patients and location of the GP office, we are confident to generalize our results to the other GP based health care system outside Switzerland. Residual confounding is a limitation. Our data set did not include data on patient symptoms or health care outcomes. It would have been interesting to stratify our results on different groups by e.g. symptoms or healthcare need. However, our results describe a general association of a GP renovation with increased patient experience.

### Comparison with existing literature

Although only few studies are available, they share a common finding that perceptual bias most likely is responsible for an improvement in patient experience in domains that were not affected by a renovation. Rice G. et al. [15] studied about 1,000 patient questionnaires after changing a GP office in Bristol, UK. They showed a similar improvement of patient experience probably secondary to a positive influence on staff’s motivation and anxiety of patients, areas that we did not measure in our study. But they also increased the size of the consultation rooms, which is known to positively affect communication [20], adapt the sitting comfort and seating patterns in consultations rooms which are also known to affect positively the communication [20]. These differences might partly explain their findings. The improvement from room modernization on patient experience was also found in hospital settings such as a study by Swan J. et al. [21] in the US. The best way to explain the reason for the perceptual bias was illustrated by DeLia D. *et al*. [22] using a written survey asking patients from 22 outpatient clinics about which aspects had most influence on patient experience. They found that office appearance and the personal treatment by the physician were the most important contributors to patient experience. Patient experience, however, can also be affected by other changes to a GP office such as clothing of GPs, which was not done so in our study. Petrilli CM. et al. [23] reviewed the role of the physician appearance, which was found to be associated with patient perceptions of trust and confidence. They proposed that studies targeting the appearance of the GP and GP office should represent the next logical step in improving patient satisfaction. Asprey A. *et al*. [24] suggested that confounding factors such as perceptual bias should be investigated if there are any doubts on the result of a GP Patient Survey (GPPS). Therefore, the core group members of European Task Force on Patient Evaluation of General Practice Care (EUROPEP) suggested to have questions about the state of the office (cleanness, periodicals, brochures, general impression) in the GPPS, but this was as yet not systematically introduced. In contrary, in 2014 the NHS removed questions about the design of the reception in their GPPS [1].

### Implications for research and practice

However, our findings implicate a different strategy for the use questions about office appearance in a GPPS. In line with the EUROPREP recommendation we suggest to take the patient’s environment into account when measuring patient experience in primary care: either by adjusting future studies for the time since last renovation or by restricting measurement in patient experience to at least 1 year after the last renovation to avoid positive confounding of renovation on other domains of quality of care. For further studies, the possible association of patients’ environment and clinical outcomes should also be studied such as done by Ilanwarne *et al*. [25]. When measuring patient experience in >7000 GP offices in the UK they found a positive association of high patient experience with better outcomes in all the Quality and Outcomes Framework. Although they found significant associations, the strength of the associations was weak. The psychological mechanism which influence patient experience has also to be better understood, as suggested by Rice G. et al. [15]. We need to explore if renovations also improve moods and performance of medical professionals.

### Conclusions

Interior renovation of a GP office probably causes a perceptual bias for >1 year that improves how patients rate quality of care. This bias could be reproduced after a second renovation strengthening a possible causal relationship. These findings imply to appropriately time measurement of patient experience to at least one year after interior renovation of GP practices to avoid environmental changes influences the estimates when measuring patient experience.

## Supporting information

S1 Appendix (DOCX)

S1 Dataset (XLSX)
