# Supplementary material for: Interior renovation of a general practitioner office leads to a perceptual bias on patient experience for over one year
Source: PLoS One. 2018 Feb 20;13(2):e0193221. doi: 10.1371/journal.pone.0193221 (PMC5819831; doi:10.1371/journal.pone.0193221)
Supplement: S1 Appendix — (DOCX) [file pone.0193221.s001.docx]

**S1 Appendix. Questionnaire**

**Survey study on patient experience**

**Date: ______________**

**Year of birth:** ______________

**Sex**: female male

**Which is your responsible GP?** ___________________**_____________**

**For how long are you with this GP?** < 2 months

< 1 year

1-5 years

>5 years

**Please rate the items below using these marks**

**(1 = very poor, 6 = very good):**

**Appearance of the office**

1. Appearance of the facility

1

2

3

4

5

6

1. Diagnostic equipment

1

2

3

4

5

6

1. Level of hygiene

1

2

3

4

5

6

1. Prompt response to patient needs

1

2

3

4

5

6

1. Punctuality and dependability of the staff

1

2

3

4

5

6

**Qualities of the medical assistant**

1. Dress and grooming of the medical assistants

1

2

3

4

5

6

1. Friendliness and courtesy of the medical assistants

1

2

3

4

5

6

**Qualities of the general practitioner (GP)**

1. Attentiveness and responsiveness of the GP to patient needs

1

2

3

4

5

6

1. GP’s level of expertise

1

2

3

4

5

6

1. GP’s level of empathy

1

2

3

4

5

6

**General satisfaction**

1. Medical performance of the GP office in general

1

2

3

4

5

6

1. Overall satisfaction with the office

1

2

3

4

5

6

**General remarks**

**We thank you for your participation**
